# Supplementary figures and images for: Specific interaction of an RNA-binding protein with the 3′-UTR of its target mRNA is critical to oomycete sexual reproduction
Source: PLoS Pathog. 2021 Oct 14;17(10):e1010001. doi: 10.1371/journal.ppat.1010001 (PMC8547697; doi:10.1371/journal.ppat.1010001)

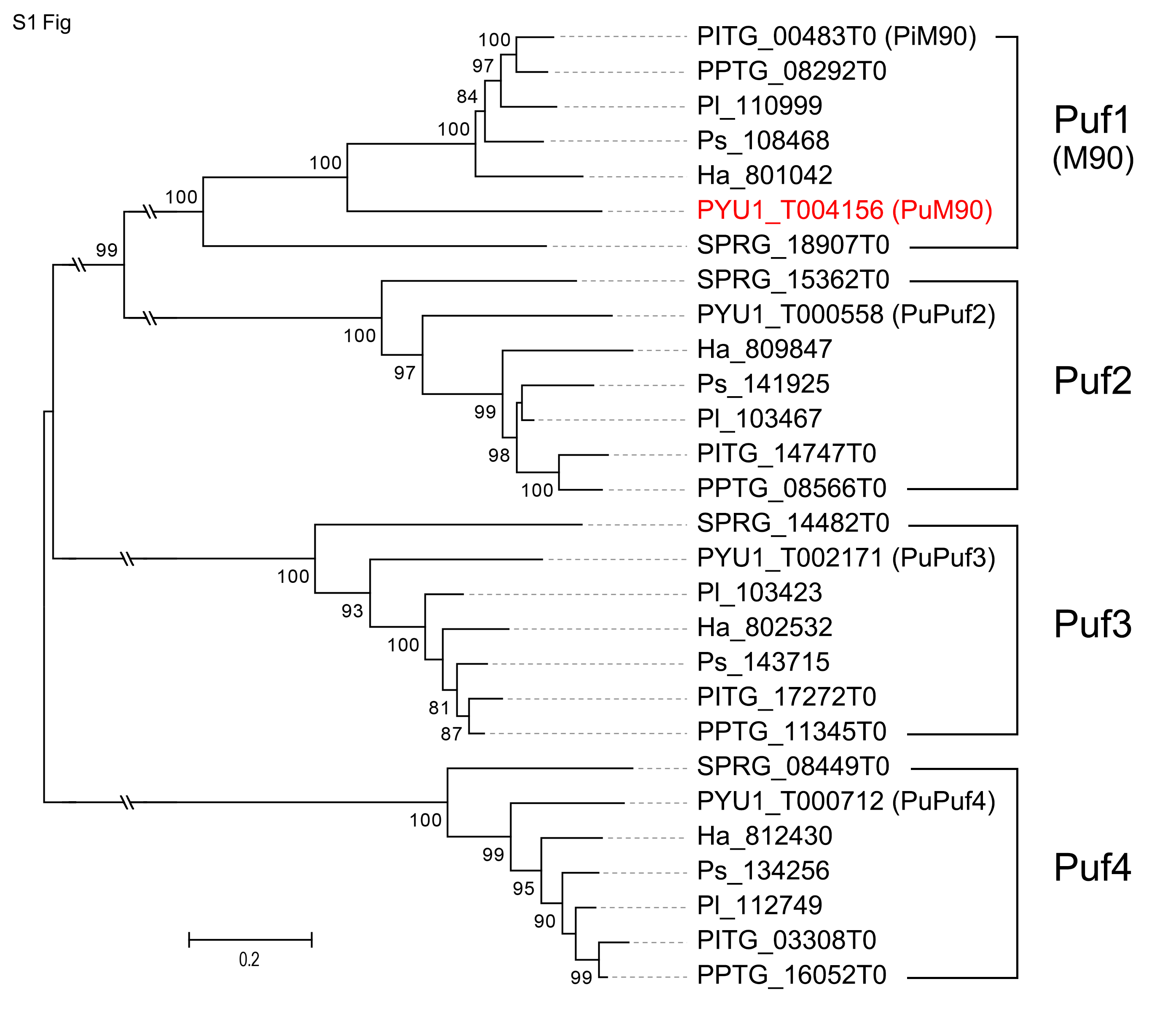

Supplement: S1 Fig — The phylogenetic trees were constructed using neighbor-joining method with 1,000 bootstrap replicates in MEGA 7.0 software. Bootstrap values higher than 80 are displayed. (TIF) [file ppat.1010001.s001.tif]

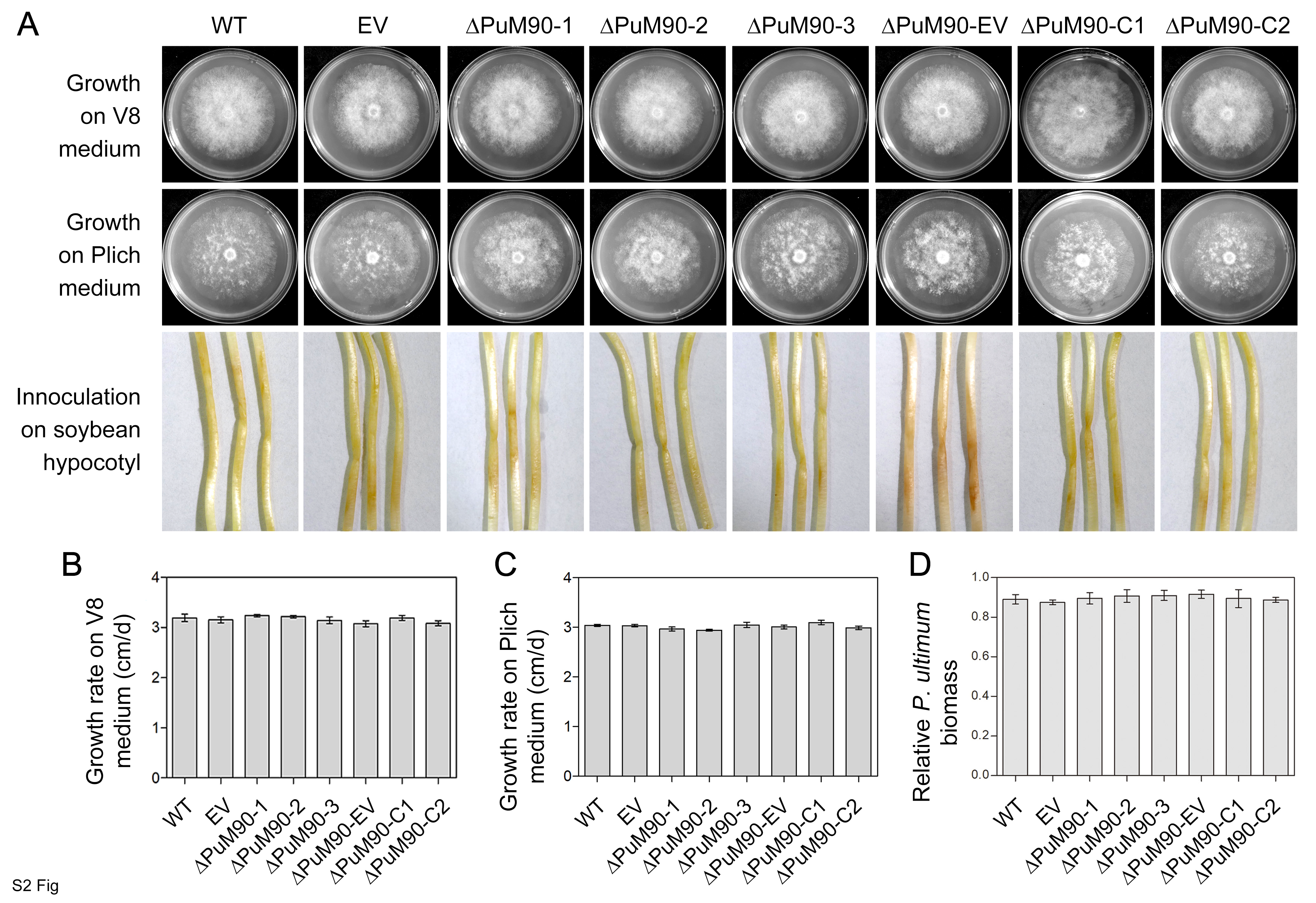

Supplement: S2 Fig — (A) Growth characteristics at 24 h after inoculation on V8 medium and Plich medium, and virulence on soybean hypocotyls of WT, EV, PuM90-knockout transformants (ΔPuM90-1/2/3), complemented transformants (ΔPuM90-C1/2), and the empty control line of ΔPuM90 (ΔPuM90-EV). (B, C) Growth rates on V8 medium (B) and Plich medium (C). (D) Relative P. ultimum biomass detected through qRT-PCR at 24 h after hypocotyl infection. (TIF) [file ppat.1010001.s002.tif]

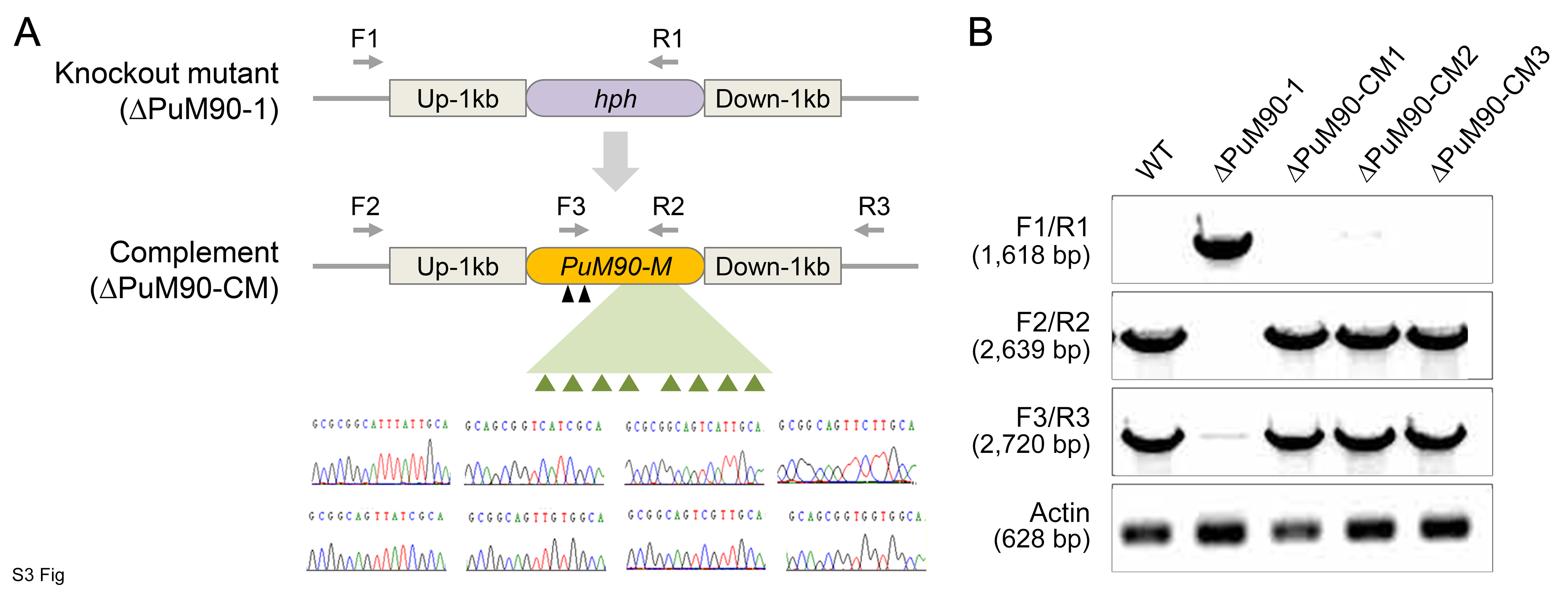

Supplement: S3 Fig — (A) Locations of the primers used to screen for complemented transformants (top) and Sanger sequencing traces of the mutated sequence regions in all eight Pumilio repeats (bottom). (B) Analysis of genomic DNA using the primers shown in (A) and actin primers as a positive control. (TIF) [file ppat.1010001.s003.tif]

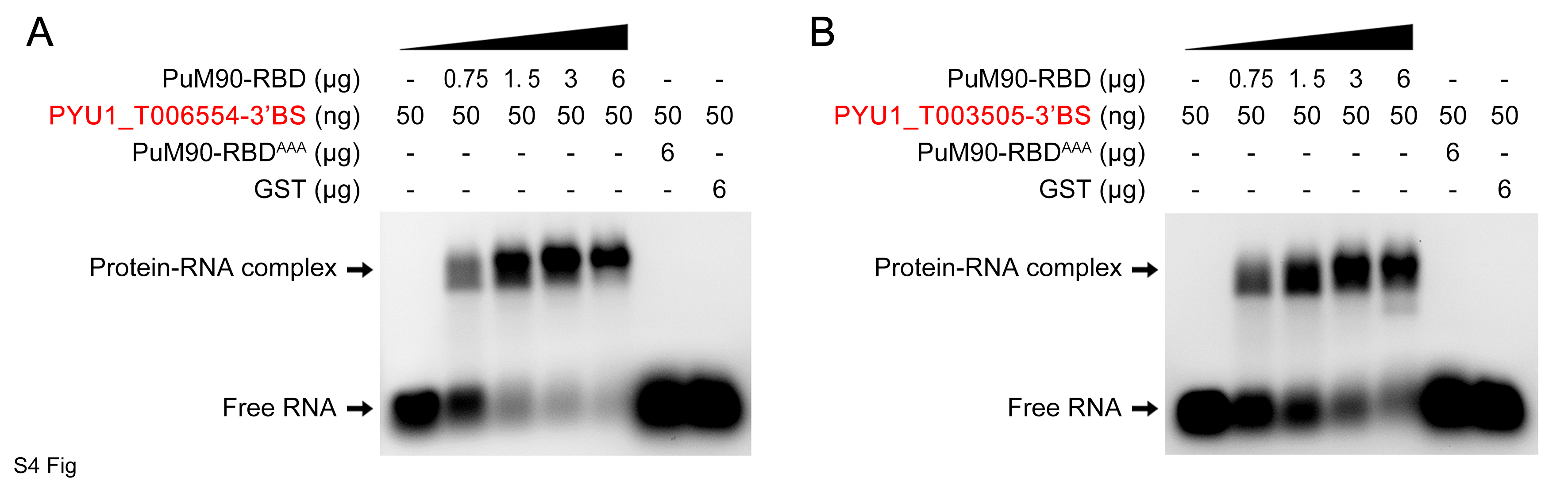

Supplement: S4 Fig — EMSA results showing that PuM90-RBD bound to the 3′BS of PYU1-T006554 (A) and PYU1-T003505 (B), while the mutant PuM90-RBDAAA and GST did not. (TIF) [file ppat.1010001.s004.tif]

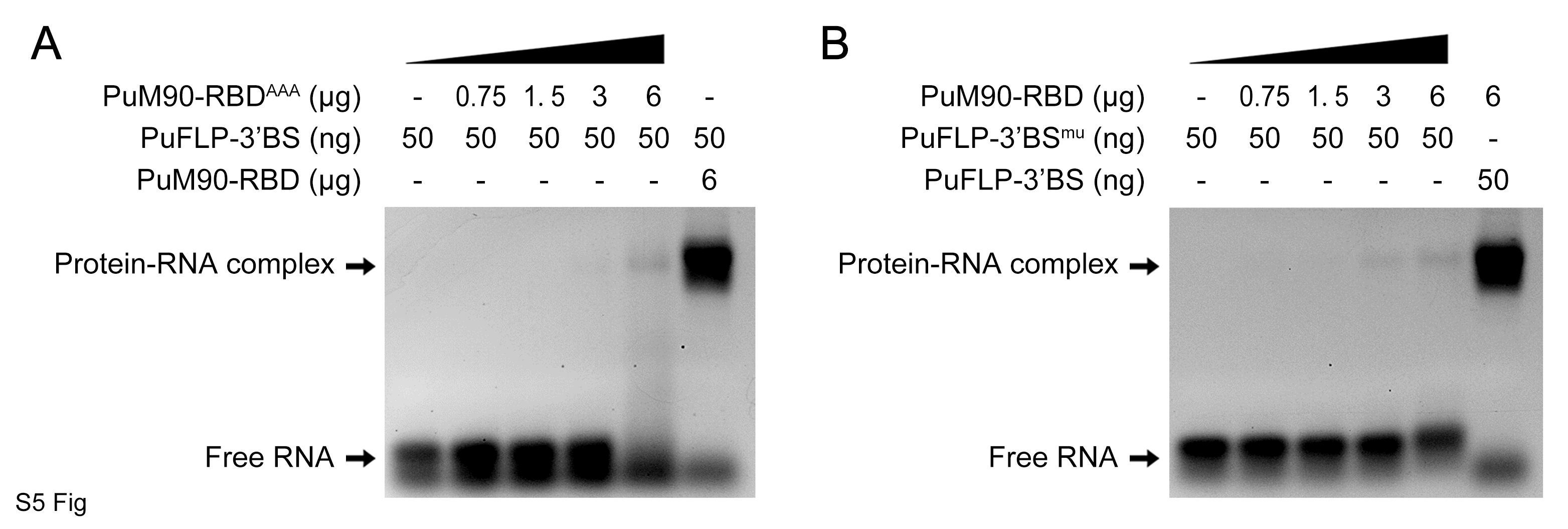

Supplement: S5 Fig — (A) The PuM90-RBDAAA peptide nearly abolished detectable binding to PuFLP-3′BS. (B) The PuM90-RBD peptide nearly abolished detectable binding to PuFLP-3′BSmu but formed a weak RNA–protein complex at a high PuM90-RBD concentration. (TIF) [file ppat.1010001.s005.tif]

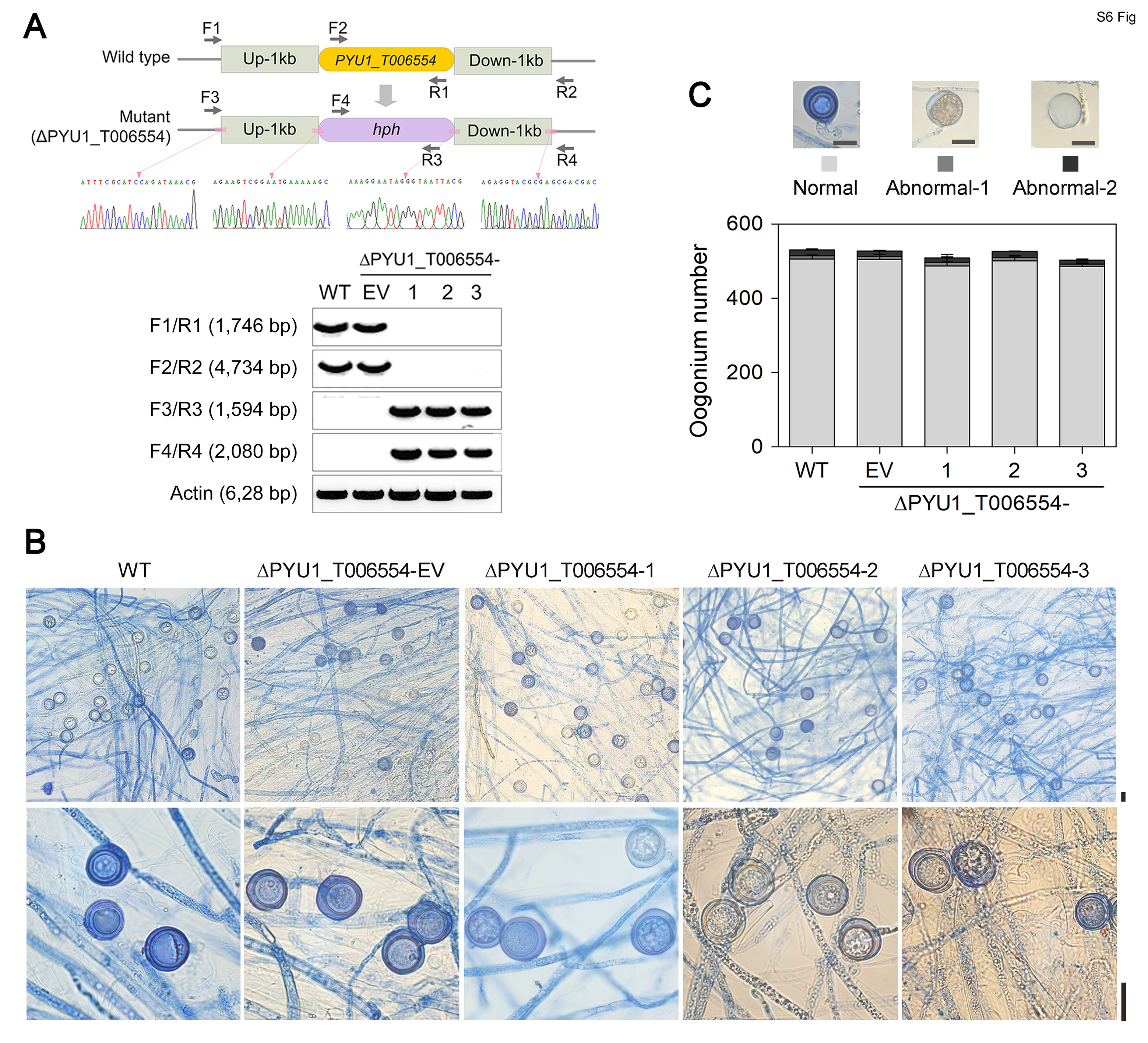

Supplement: S6 Fig — (A) Construction and verification of three representative candidate mutants for PYU1_T006554 knockout. (B, C) Morphology (B) and number (C) of oogonia generated in 14-day-old cultures. Bar, 20 μm. (TIF) [file ppat.1010001.s006.tif]

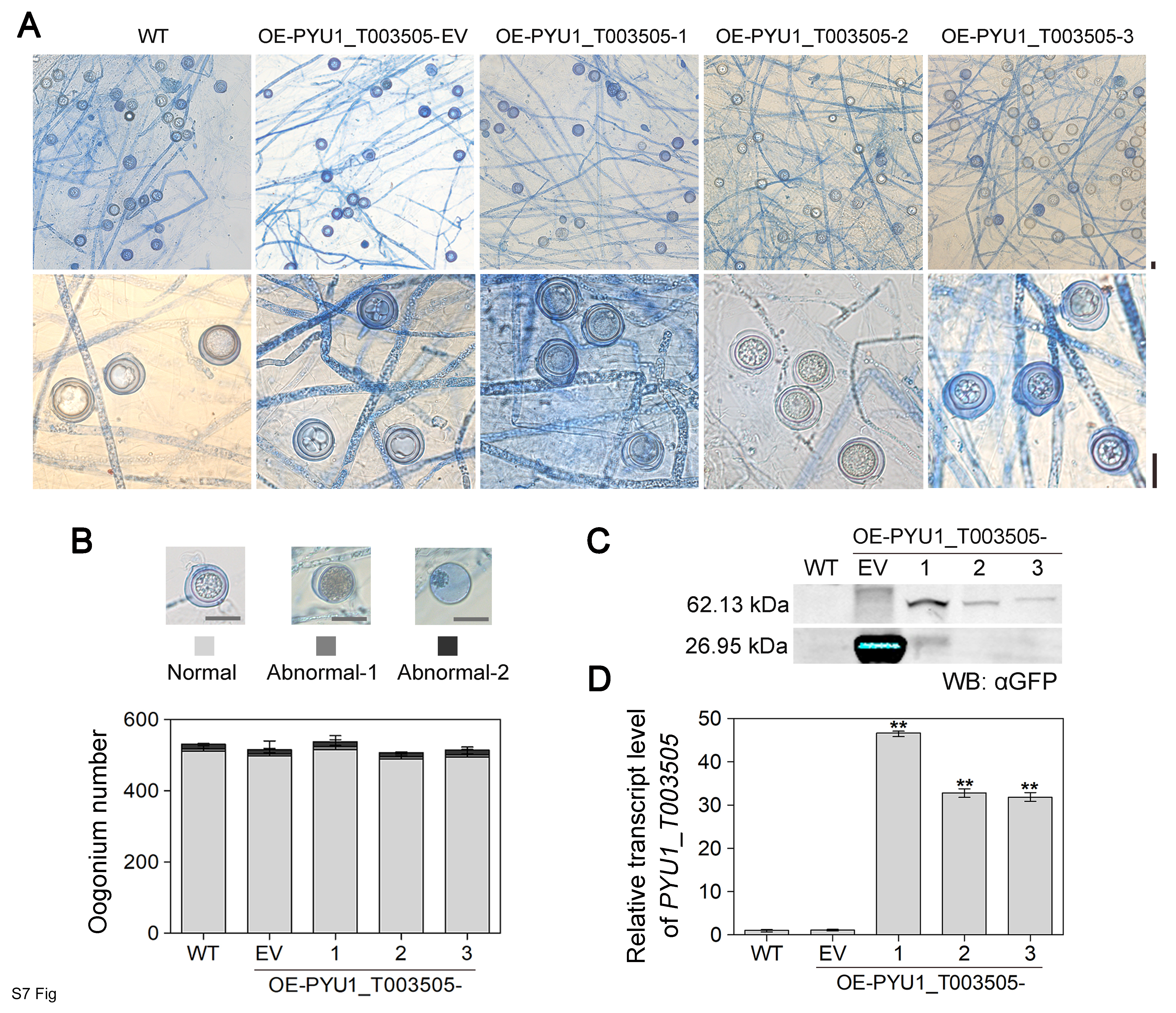

Supplement: S7 Fig — (A, B) Morphology of oogonia and oospores (A) and number of oogonia (B) generated in 14-day-old cultures. Bar, 20 μm. (C) PYU1_T003505 protein levels measured through Western blotting. (D) PYU1_T003505 transcript levels measured through qRT-PCR. Asterisks (**) indicate significant differences comparing with WT at P < 0.01. (TIF) [file ppat.1010001.s007.tif]

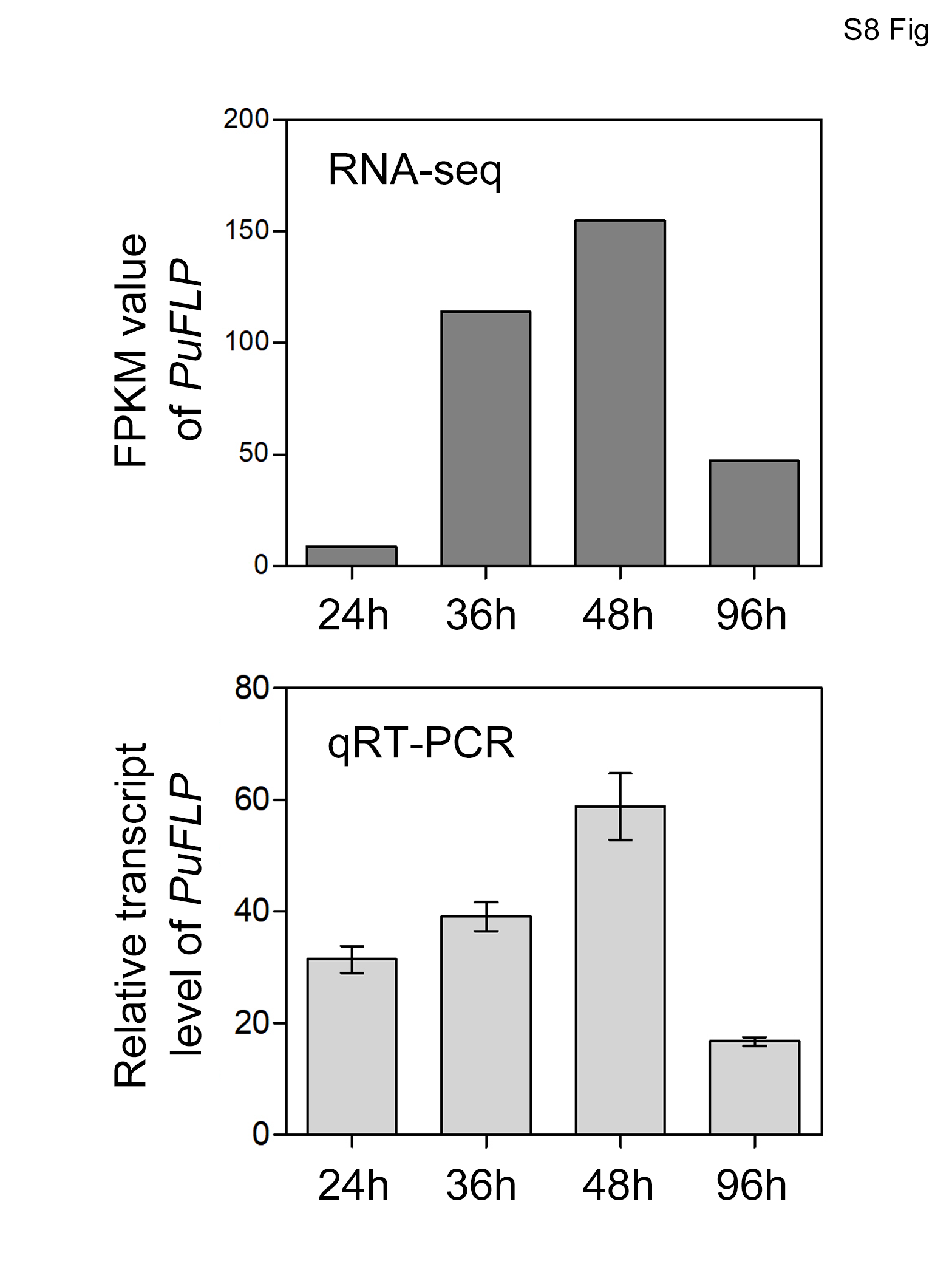

Supplement: S8 Fig — Transcript levels of the PuFLP genes measured using RNA-seq (top) and qRT-PCR (bottom) when P. ultimum mycelia were cultured in V8 liquid medium for 24, 36, 48, or 96 h. (TIF) [file ppat.1010001.s008.tif]

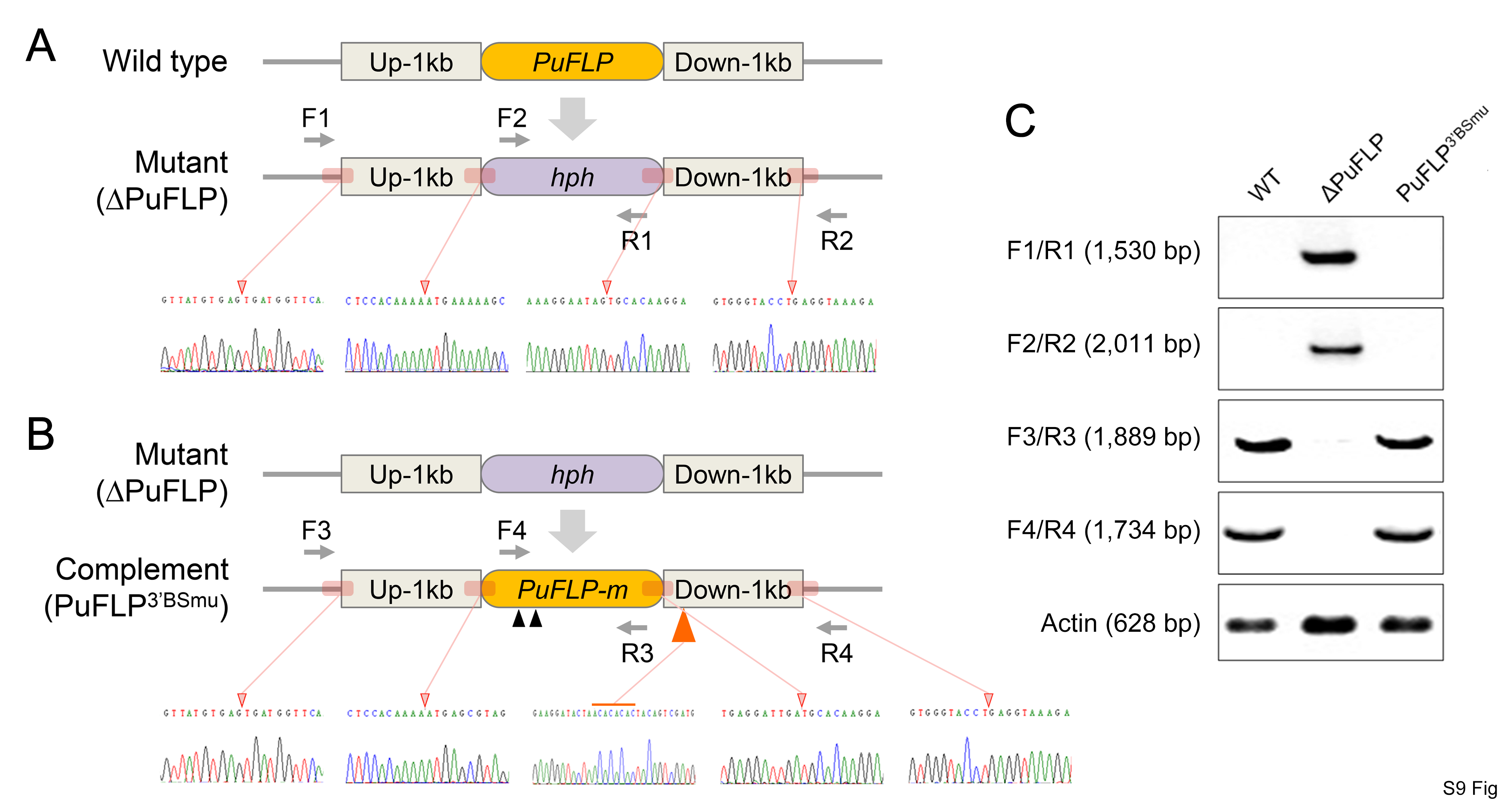

Supplement: S9 Fig — (A) Construction and verification (with Sanger sequencing traces) of the PuFLP-knockout mutant (ΔPuFLP). (B) Construction and verification (with Sanger sequencing traces) of the complementation line PuFLP3′BSmu, in which the UGUACAUA motif in PuFLP-3′BS was replaced with ACACACAC. (C) Analysis of genomic DNA PCR products using the primers shown in A and B and actin primers as a positive control. (TIF) [file ppat.1010001.s009.tif]
