# Supplementary material for: Specific interaction of an RNA-binding protein with the 3′-UTR of its target mRNA is critical to oomycete sexual reproduction
Source: PLoS Pathog. 2021 Oct 14;17(10):e1010001. doi: 10.1371/journal.ppat.1010001 (PMC8547697; doi:10.1371/journal.ppat.1010001)
Supplement: Attachment — Submitted filename: Response letter-20210917.pdf [file ppat.1010001.s013.pdf]

2021-9-17

Dear PLOS Pathogens editors and reviewers,

Thanks for handling and reviewing the manuscript and providing very good suggestions and detailed comments for improving it. According to these, we have carefully revised the manuscript.

In this letter, we describe our detailed responses to the suggestions or questions (in order to facilitate reading, some similar questions about simple revisions were responded together). We also uploaded a version of the revised manuscript with red font denoting where the text has been changed.

Thank you.

Sincerely,

Dr. Wenwu Ye
Associate Professor
Department of Plant Pathology
Nanjing Agricultural University
Nanjing, China 210095
TEL: (+86) 025-84399621
Email: yeww@njau.edu.cn

Dear Dr. Ye,

Thank you very much for submitting your manuscript "Specific interaction of an RNA-binding protein with the 3′-UTR of its target mRNA is critical to oomycete sexual reproduction" for consideration at PLOS Pathogens. As with all papers reviewed by the journal, your manuscript was reviewed by members of the editorial board and by several independent reviewers. The reviewers appreciated the attention to an important topic, and I especially was pleased to see molecular genetic studies being applied to *Pythium*.

I do not believe that additional experimentation is required for the paper, even though reviewer #1 has made suggestions for additional experiments. Nevertheless, each reviewer has made important suggestions for improving the manuscript. We are likely to accept this manuscript for publication, providing that you modify the manuscript according to the reviewers' recommendations.

Please prepare and submit your revised manuscript within 30 days. If you anticipate any delay, please let us know the expected resubmission date by replying to this email.

When you are ready to resubmit, please upload the following:

[1] A letter containing a detailed list of your responses to all review comments, and a description of the changes you have made in the manuscript.

Please note while forming your response, if your article is accepted, you may have the opportunity to make the peer review history publicly available. The record will include editor decision letters (with reviews) and your responses to reviewer comments. If eligible, we will contact you to opt in or out

[2] Two versions of the revised manuscript: one with either highlights or tracked changes denoting where the text has been changed; the other a clean version (uploaded as the manuscript file).

Important additional instructions are given below your reviewer comments.

Thank you again for your submission to our journal. We hope that our editorial process has been constructive so far, and we welcome your feedback at any time. Please don't hesitate to contact us if you have any questions or comments.

Sincerely,

Howard S. Judelson, Guest Editor

Bart Thomma, Section Editor

Kasturi Haldar, Editor-in-Chief

Michael Malim, Editor-in-Chief

Reviewer Comments:

Part I - Summary
Reviewer #1:

In this manuscript, the authors describe the identification and characterization of RNA binding protein called PuM90. This protein is found within *Pythium ultimum* and supposedly has orthologs within other oomycetes, such as *Phytophthora infestans*. The authors describe how PuM90, through its Pumilio containing domains, bind the 3' UTR of another gene called PFLP and negatively regulates its expression.

The authors hypothesize that the formation of oospores is regulated through PuM90 activity and its consequent modulation of *PuFLP* expression. Overall, the manuscript is well written and presents high-quality data. I am particularly impressed with the authors' genetic analyses, using CRISPR/CAS9 and complementation assays to validate PuM90 functionality.

The authors demonstrate that PuM90 is a gene induced during oospore formation and that it encodes a protein with Pumilio motifs. The authors identify four different Pumilio domain-containing proteins, each of which has different number of RBP domains and predicted length.

Unfortunately, the authors do not show what other domains and configurations are present within this protein family (is it a family?). It is an important question as the length and composition of these proteins seems to be very diverse.

R1: We provided the sequence and domain structure information of the Puf proteins identified in *P. ultimum* and some other representative oomycetes in **S1 Table**, and added **S1 Fig** which shows the phylogenetic relationship among the Puf proteins. We also added the related description and discussion in the manuscript.

In brief, there are four members of Puf protein family in each analyzed oomycete species. Each member shows a clear 1:1 orthologous relationship (in phylogenetic analysis), as well as similar sequence length and domain structure among oomycetes; but different members are diverse.

Further comparative analysis to learn functional conservation and/or diversity among the Puf proteins in oomycetes would be interesting.

Next, the author's look at the transcription level of *PuM90*, and do so by using RNA-Seq of RNA derived from mycelia. Whilst it's exciting and beneficial to identify genes associated with sporulation, much of the work presented was not done *in vivo* (during infection). For example, the authors generated PuM90 mutants and complementation mutants and assessed their impact on sporulation *in vitro* and *vivo*. Equivalent *in vivo* work for PuFLP is not presented.

R2: As far as we know, PuM90 specifically functions on the sexual development of *P. ultimum*, thus we focus on this biological process in this study.

Given that PuM90 is predicted to be an RNA binding protein, the authors identified possible binding partners or target RNAs. They did so by using the modularity of the PuM90, which allows them to predict the RNA sequence motif PuM90 would bind. In doing so, and through computational analysis and gene expression analysis, they identified three strong candidates that seemed to be regulated.

The authors then used RNA binding essays to demonstrate that PuM90 can bind to the 3' UTR of these three transcripts and that by removing or modifying PuM90 sequence-specificity, binding is limited or reduced. Unfortunately, what the authors did not show whether this was sequence-specific. It would have been informative to mutate the 3' UTR binding motif and see whether that impacts on binding in these essays. Alternatively, the authors could have selected a UTR for a transcript that they do not expect PuM90 to target.

R3: In addition to the mutations in the RNA-binding domain of PuM90 (EMSA: PuM90-RBD^{AAA} peptide; *P. ultimum* mutants: △PuM90-CM1/2/3), we also mutated the 3' UTR binding motif of *PuFLP* using both *in vitro* (EMSA: PuFLP-3'BS^{mu} fragment) and *in vivo* (*P. ultimum* mutants; PuFLP$^{3'BSmu}$) assays and the results have supported a sequence-specific interaction (**Fig. 6**, **S3**, and **S4**).

One critical experiment in demonstrating a direct relationship between PuM90 and PuFLP1 is to assess phenotypes and relationships between mutations. In this regard, the question as to whether overexpression of *PuFLP* phenocopies *PuM90* deletion is important. Indeed, the authors show that overexpression of *PuFLP* leads to reduction of oospore formation. These results strongly suggest that PuM90 acts on PuFLP transcripts, and by extension, regulates oospore formation. What is somewhat surprising, however, is that deletion of *PuFLP* returns a wild-type phenotype. Given that this protein inhibits oospore formation, one would expect some sort of a developmental phenotype. It also begs the question: what would a PuM90/PuFLP double mutant phenotypically look like? The actual mechanism(s) may be a little more intricate as the presented model would suggest. If both PuM90 absence and high levels of PuFLP are required for oospore development to be perturbed, one could think that the product(s) arising from PuFLP processing (prompted by PuM90) has a role in oospore formation. A double mutant may clarify this point in the future.

R4: To learn if the UGUACAUA motif of the *PuFLP* 3′-UTR was essential for PuM90 binding, we performed *in situ* complementation strategy (**S8 Fig**) to generated *P. ultimum* mutants in which the UGUACAUA motif was substituted with ACACACAC (PuFLP$^{3'BSmu}$) and *PuFLP*-complemented transformants (ΔPuFLP-C) as control. ΔPuFLP-C, rather than *PuFLP*-deletion mutants return a wild-type.

The phenotypes of *PuFLP*-deletion mutants were previously not mentioned, because we aimed to explain why deletion of *PuM90* resulted in a higher transcript level of *PuFLP*. Actually, the phenotypes of *PuFLP*-deletion mutants were weaker but similar to those of the *PuFLP*-overexpression mutants. We added a description in the discussion part.

Comparing with *PuM90*, the transcript level of *PuFLP* is induced earlier (**S7 Fig**), thus we speculated that *PuFLP* may function at an earlier stage of sexual development, although deletion of *PuFLP* finally also affect oospore formation. In this study, we just proved that non-repression of *PuFLP* in the later stage (96 h; PuM90 regulates) is not conducive to oospore formation. More detailed assays are needed to learn and explain the functions of PuFLP itself in the future.

Overall, the data itself and in the way it is presented, is convincing and strongly supports a model suggestive of a new mechanism for gene regulation in *Pythium*. However, there are several outstanding questions that I think would dramatically increase the impact and importance of this work.

Firstly, the author states that PuM90 is an ortholog of *Phytophthora infestans*. However, no such evidence is presented. How many M90-like proteins are there in *P. infestans* and *P. ultimum*? If there are more than one, are they true orthologs?

Please see R1.

Secondly, the authors suggest and invoke a mechanism that may be conserved across the oomycetes. It would be particularly interesting to understand protein family composition across the oomycetes and to know whether there's any evidence of expansion or deletion in specific lineages. For example, what would regulation look like in pathogens that produced other spore types? Providing a couple of hints, through some rather basic analyses, would raise the interest of this work considerably.

R5: It is a good question. That needs a series of functional assays for many more Puf genes in different oomycetes, and a comprehensive analysis between pathogen biology and gene phylogeny and functions. As mentioned in R1, we add some related data as well as a special discussion to describe this family in the discussion part.

Reviewer #2:

The manuscript by Feng et al. reports the identification a Puf family RNA-binding protein, PuM90, as an important player in *Pythium ultimum* oospore formation, and the underlying post-transcriptional regulatory mechanism. The Puf domain of PuM90 specifically binds to a UGUACAUA motif in the mRNA 3′ untranslated region (UTR) of PuFLP, a flavodoxin-like protein, and thereby downregulate PuFLP expression to facilitate oospore formation. This study not only sheds important insight into the regulation of sexual reproduction of *P. ultimum*, but also reports CRISPR/Cas9 system-mediated gene knockout and *in situ* complementation methods for *Pythium* for the first time. *P. ultimum* is a homothallic oomycete and sexual reproduction plays an important

role in its life cycle. The identification of key components in oospore formation is significant as these components can serve as potential targets of disease control. This study is well designed and well executed. The manuscript is concise and well written overall. However, some parts may need be more detailed to be readily understandable by the readers.

Reviewer #3:

This MS focusses on a RNA-binding protein in the oomycete plant pathogen *Pythium ultimum* that is named PuPuf1 aka PuM90. The authors nicely show that transformants lacking this protein show defects in oospore formation and they unravel how a domain in PuM90 that carries Pumilio repeats targets a short region in the 3'UTR of mRNAs thereby repressing translation of those mRNAs. One of the target genes that is identified is PuFLP. It encodes a flavodoxin-like protein and transformants overexpressing PuFLP are phenocopies of the PuM90 knock-out transformants. The MS is a pleasure to read and the research is technically sound. I have a few questions and comments, and a list of items to need to be considered when revising the MS.

Part II – Major Issues: Key Experiments Required for Acceptance
Please use this section to detail the key new experiments or modifications of existing experiments that should be absolutely required to validate study conclusions. Generally, there should be no more than 3 such required experiments or major modifications for a "Major Revision" recommendation. If more than 3 experiments are necessary to validate the study conclusions, then you are encouraged to recommend "Reject".

Reviewer #1: Suggested experiments listed above (though they do not stand in the way of publication in my view)

Reviewer #2: (No Response)

Reviewer #3: (No Response)

Part III – Minor Issues: Editorial and Data Presentation Modifications
Please use this section for editorial suggestions as well as relatively minor modifications of existing data that would enhance clarity.

Reviewer #1: Some minor editorial issues (Western blot with a capital 'w').

R6: Corrected.

Reviewer #2: All the primers are listed in Table S3. Although short notes are included under "Application", in some cases it is difficult to figure out how these primers were used. The authors should add more detailed description in the table or table notes, associate the names better with the main manuscript, differentiate sgRNA target sequences and gene specific sequences from added sequences for cloning purpose. The sgRNA target sequences should be also included in the main manuscript. Fig. 2, The primers in A and B are very confusing. F2/R2 seems to be the same in both panels. How about others? In Table S3, there seems to be a set from F1/R1 up to F4/R4. However, the primers in both panels do not correspond. The authors need clarify and label them clearly.

R7: We updated **S3 Table** to provide more detailed description of the primers and sgRNAs used in this study. We also added fragment size of amplification product for each pair of primers in the figures.

For all figures with transcript levels from RNA-seq and RT-qPCR, please describe what the number values represent in the legends. For example, Fig 1B, Fig 6B and more.

R8: We updated the Y-axis titles in corresponding figures to make it clearer.

For multiple figures showing statistically significant difference, the author should clearly indicate to which it the comparisons were made.

Fig. 4B, describe the red square boxes, add scale bar for the bottom panel.

Line 522, $2^{[Ct(PuACTA-Ct(GmCYP2)]}$ should be $2^{-[Ct(PuACTA)-Ct(GmCYP2)]}$. Please double check.

Line 530. Add reference for pTOR::eGFP, or describe it.

For line 191-202, Key amino acids in the TRM of the Puf domain determine PuM90 function. 24 amino acids were mutated to Alanine. This may cause the collapse of the protein structure. In this case, the conclusion is not warranted. To interpret the results more accurately, the authors may include a short discussion on this, and change "Key amino acids in the TRM of the Puf domain determine PuM90 function" to "Mutations in key amino acids in the TRM of the Puf domain compromise PuM90 function".

R9: all revised.

Reviewer #3:
One aspect that I miss in the discussion is a reflection on the potential activity of the flavodoxin-like protein, and why and how its presence disturbs oospore formation. I conclude from lines 259-260 that *PuFLP* knock-out transformants were generated but there is no description of the phenotype. Is FLP required for asexual development or for virulence? And what is the overall expression profile of *PuFLP*?

Please see R4.

Relation between PuM90 and PiM90.

Line 122-124: this is the first time in the MS that the *P. infestans* ‘ortholog’ M90 is mentioned. Is PuPuf1 really the ortholog of PiM90? There are at least two publications on M90 in *P. infestans* but only one is included as reference. That is #31 (Fabritius et al.) in which one can trace back that M stands for mating and 90 is just a random number of a cDNA clone. The authors should reconsider the name ‘PuM90’. Adapting that name requires data showing that PuPuf1 is really the Puf that is closest to PiM90. So please add a phylogenetic tree of Puf’s in *P. ultimum* and in *Phytophthora* species, also to clarify if there are additional Puf’s in *Phytophthora* apart from M90. Fabritius et al. (31) noted that it is single copy gene based on Southern blot analysis. The other paper on M90 (Cvitanich and Judelson 2003 Eukaryotic Cell) is a more in depth study showing, amongst others, that in *P. infestans* M90 is also expressed during asexual development. Expression in *P. infestans* thus differs from expression in *Pythium*. It is worth to note that, and to include a reference to Cvitanich and Judelson (2003). In the discussion also PlM90 from *Perenophytophthora litchi* is mentioned (line 275-276) (reference #37). Also this gene is expressed during asexual reproduction as well as sexual reproduction. How does PlM90 relate to PuPuf1 (PuM90) and PiM90?

R10: (1) Please see R1. (2) As mentioned in precious reports and we also tested many *P. ultimum* var. *ultimum* strains as well as conditions, thus far we failed to induce zoosporangia and zoospore production. Therefore, the expression of *PuM90* at asexual reproduction stage cannot be obtained for comparison.

Abstract. Revisit line 21 -24. Is it so that binding of PuM90 to the 3’ UTR of the mRNA causes inhibition of translation, thereby resulting in less PuFLP protein? Line 24 says ‘downregulates PuFLP expression’ which suggests interference at the level of gene expression, so at the DNA level.

R11: Revised as ‘repress *PuFLP* mRNA level’ in the full text.

Line 50-53: revisit this sentence. It is hard to read. Also, I find word ‘interestingly’ misplaced and the statement in line 53 (‘...has become more important…’) confusing. It suggests that over time, oospore production has taken over zoospore production. But is that true? Is there evidence and is this described in reference #8? Referring to reference #7 in line 53 is likely wrong. It a review paper on *Phytophthora infestans*, not on *Pythium ultimum*. Check.

Line 24: downregulates

Line 28: critical to = critical for

R12: All checked and revised.

Line 37: is reference 2 appropriate for the statement that ‘all known oomycetes can undergo sexual reproduction’? Unlikely giving the title of the paper. Please check.

R13: We revised the sentence and gave proper references.

Line 58: sensitive OR more sensitive?
Line 59: Also, *P. sojae PsGK5*-silenced mutants lost the ability to produce oospores. Refer to Yang et al. 2013 Molecular Microbiology 88, 382-394.
Line 62-63: 'In addition'? Revisit this sentence. What is meant by 'critical regulators'? Could PsYPK1 (functions as a kinase) be considered as a critical regulator?
Line 64: Publication? OR is it: 'Availability', or maybe 'release'?
Line 66: protoplast electroporation? For protoplasts PEG-mediated transformation is common. For zoospores, electroporation.
Line 67 and 72: Weiland 2003 Current Genetics showed protoplast transformation of *Pythium aphanidermatum*.
Line 67: Vijn 2003 FGB showed Agrobacterium mediated transformation of *P. ultimum*
Line 68: also in *P. infestans*. Include the reference to the recent paper (2021) of the Judelson lab in MPP.
Line 75-76: revisit this sentence. '..during RNA [……] stability….' Is not correct.

R14: All checked and revised.

Line 75: I notice that the authors consider 'regulation of gene expression' as a process that occurs at all levels. I have learned that gene expression is a process that occurs solely at the DNA level so based on that, gene expression cannot be regulated at the post-transcriptional level and (in line 84) targeted mRNAs cannot be expressed. In this line of thinking proteins cannot be expressed so in line 88 the word 'expression' is misplaced and should be replaced by, for example 'synthesis' or 'production'. Line 105-106: PuM90 acts at the RNA level so does it actually 'repress translation of *PuFLP* mRNA' rather than 'repress expression of' the gene ?
Line 90: '…downregulates translation of *Ash1p* mRNA.'

R15: All checked and revised.

Line 96: Is it more precise to write: '…it is possible to predict the specific RNA sequence to which a PUF protein binds' ?
Line 102: was = is
Line 117: '… the transcript level of *PuPuf1* was strongly increased….'
Line 121: here the authors take a too sharp corner when stating that PuPuf1 may be involved in oospore formation. One could say that 'this coincides with the increase in PuPuf1 transcript levels'.

R16: All revised.

Legend figure 1; line 746: …repeats in PUF proteins….
Line 747: Transcript levels NOT transcription levels. PUF genes OR Puf genes as the figure and in the main text? Be consistent.
Figure 1B: rephrase 'hours post [..] culturing'. That is not correct.
Figure 1C and the legend of Figure 1C: the reader has to guess what is shown here. Point out the gametangia (although I cannot distinguish gametangia in these photo's), oospores etc. Indicate in the legend how the tissue was stained.
Line 127: improved? Why improved and compared to which strategy?
In figure 2 lower panels: indicate the sizes of the PCR fragments.

Line 762: the triangles are pink colored not black.

R17: All revised.

Line 139: rephrase '..they would be capable of further transformation…'.
Line 146: how many complemented strains were obtained?
Legend Figure 3A; lines 773 and 774: first line = first row etc.
Line 774 and 781: … stained with lactophenol-trypan blue… (rather than 'treated')

R18: All revised.

For the data shown in Figure 3B and 3C it is not clear how oospore diameter and wall thickness, respectively, was determined. How was wall thickness measured in the photographs in Figure 3A? line 177-178 suggests the TEM is confirming the difference in thickness implying that the original measurements were based on bright field images.

R19: We used microscope graticules to measure oospore diameter and wall thickness. According to the specimen size observed by the eyepiece graticules, the true size of the specimen can be obtained by converting the data measured by the stage graticules. We added this method to the M&M part.

Line 161: figure 3B shows total number of oospores, not oogonia. Check.

R20: Corrected.

Line 169: '… was investigated in mycelium cultured for 2, 4 and 7 days in V8 broth.'
Line 193-193: revisit this sentence. It reads as if the PuM90 protein was predicted to organize into a typical crescent-shaped structure but this is limited to the domain carrying the repeats.

R21: All revised.

For Figure 5D and 5E same question as for Figure 3: it is not clear how oospore diameter and wall thickness, respectively, was determined. How was wall thickness measured?

Please see R17.

Figure 5A: mutantion = mutation

R22: Corrected.

Line 201–202: was there an attempt to distinguish between repeats? Do all repeats have to possess the three essential amino acids for proper functioning of PuM90 in oospore development? Consider to rephrase this sentence. When first reading it I was stuck by 'THESE three amino acids' whereas it is actually 3 x 8 amino acids.

Line 208 -210: Suggestion for rephrasing: By minng/analysing the 3' UTR sequences (i.e. 100 nt after the stop codon) of all predicted (?) *P. ultimum* genes, we identified 117 genes that contain this motif in their 3' UTR.
Line 213: 'transcript level' OR 'higher expression level' but NOT transcription level
Line 210-213: revisit this sentence. The formulation is too complicated.
Figure 6A: terminator codon = stop codon
Figure 6B: Y-axis 'transcript level' NOT transcription level. Also change this the legend. And check throughout the MS. See for example line 240.
Heading Table S2: … knocked out….
Table S2: Explain what is shown in each column in a text block next to the table or as notes between title and actual table.

R23: All revised.

Line 215-216: any further information about the other two genes? And why was 13662 chosen for further study and not the other two?

R24: *PYU1_T006554*-knockout (S6 Fig; failed to be overexpressed) and *PYU1_T003505*-overexpression (S7 Fig) mutants didn't show any significantly different phenotypes during oospore formation, thus they were considered as non-targets of PuM90, and not selected for further analysis.

Line 222: binding site (not sites)
Line 241: protein level – delete 'expression'

R25: All revised.

Fig S6: the overexpression of *PYU1_T003505* in *P. ultimum* is accomplished by introduction of a GFP fusion construct. Is it possible that the GFP tag interferes with the function of PYU1_T003505 and that therefore overexpression does not show a change in phenotype?

R26: pTOR::eGFP is often used in oomycetes for gene overexpress, and previous studies have shown that a GFP fusion did not interfere the function of the targets.
For example: Anna KA, Johan F, Ramesh RV, Christina D. *Phytophthora infestans* Argonaute 1 binds microRNA and small RNAs from effector genes and transposable elements. New Phytologist. 2016; 211(3): 993-1007.

Line 283: why 'unique'?

R27: Removed.

Line 285: the drawbacks are not specific for *Pythium* but more general for application of the RNAi

strategy in any organism
Line 324: delete ‘sequence’
Line 332: … can bind directly to mRNA…
Line 354: replace ‘, and’ by ‘;’

R28: All revised.

Line 368: is there a code for the WT strain? It makes sense to give it a code as this particular strain is proven to be amenable for CRISPR-Cas9 genome editing. And also as the source of the RNA seq data.

R29: We added the code F18-6 for the *P. ultimum* WT strain. The RNA-seq data has been available in NCBI database (BioProject ID: PRJNA540115).
