# Supplementary material for: Specific interaction of an RNA-binding protein with the 3′-UTR of its target mRNA is critical to oomycete sexual reproduction
Source: PLoS Pathog. 2021 Oct 14;17(10):e1010001. doi: 10.1371/journal.ppat.1010001 (PMC8547697; doi:10.1371/journal.ppat.1010001)
Supplement: Attachment — Submitted filename: Response letter-20210930.pdf [file ppat.1010001.s014.pdf]

2021-9-30

Dear PLOS Pathogens editors and reviewers,

Thanks for carefully reviewing the manuscript and providing very good suggestions for improvement of it. According to these, we have carefully revised the manuscript.

In this letter, we describe our detailed responses to the comments one by one. We also uploaded a version of the revised manuscript with red font denoting where the text has been changed.

Thank you.

Sincerely,

Dr. Wenwu Ye
Associate Professor
Department of Plant Pathology
Nanjing Agricultural University
Nanjing, China 210095
TEL: (+86) 025-84399621
Email: yeww@njau.edu.cn

Dear Dr. Ye,

Thank you very much for submitting your manuscript "Specific interaction of an RNA-binding protein with the 3′-UTR of its target mRNA is critical to oomycete sexual reproduction" for consideration at PLOS Pathogens. As with all papers reviewed by the journal, your manuscript was reviewed by members of the editorial board and by several independent reviewers. The reviewers appreciated the attention to an important topic. Based on the reviews, we are likely to accept this manuscript for publication, providing that you modify the manuscript according to the recommendations shown below:

The authors have generally provided satisfactory changes to the manuscript that address the reviewer's main concerns. A few small improvements are recommended, however:

Line 87: "not effectively applied" seems the wrong thing to say. The implication is that the methods are not effective, but I see no evidence of this. The only thing that can be said that for sure is that few people have reported using those methods. This should be reworded. Otherwise, it looks like the authors are insulting the prior work.

R1: We are sorry for the wrong word and reworded it.

Line 81: the meaning of "related genes" is unclear

R2: We revised the sentence.

Line 89: Cas9 has not been used successfully in *P. infestans*, only Cas12a has been used. You could reword this sentence to say CRISPR/Cas instead of CRISPR/Cas9.

R3: Revised.

Line 231: "100 nt after stop codon": surely these were not always exactly at this site. Do you mean within 100 nt of the stop codon? I would reword this, and perhaps indicate how far the site was on average from the polyA tail.

R4: Yes, it is “within 100 nt after the stop codon”, rather than exactly at certain site. We revised it.

Table S2: The headings could be improved to indicate that 24, 48, 96 hr etc. are wild-type

R5: Revised.

Line 234: The wording "over 12-fold more" was confusing. This is just a language issue; there were 12-fold more genes differentially expressed at the 96 hr compared to the 24 hr timepoint. Also, why not just give (or also give) the number of genes?

R6: Revised.

Finally, reviewer 3 suggested that in Fig S6 that the GFP tag interferes with the function of PYU1_T003505 in the transformant and that therefore overexpression does not show a change in phenotype? In rebuttal, the authors give a reference showing that GFP did not interfere with argonaute function in another paper. However, the effect of the tag can be protein-specific; sometimes it has no effect, sometimes it does. I do not expect the authors to perform the type of experiment typically used to address this (expressing GFP from both the C and N-termini). However, they should acknowledge that their conclusion assumes that GFP is not affecting the function of the protein.

R7: We added "assuming that the GFP tag was not affecting the function of the protein" following the description of *PYU1_T003505* overexpression phenotype.

*We also updated "S2 Fig"; there was a mistake when we were calculating the growth rate of mycelia on V8/Plich medium. The updated data do not alter the conclusion.
